# Impact of Refugees on Local Health Systems: A Difference-in-Differences Analysis in Cameroon

**DOI:** 10.1371/journal.pone.0168820

**Published:** 2016-12-16

**Authors:** Lambed Tatah, Tefera Darge Delbiso, Jose Manuel Rodriguez-Llanes, Julita Gil Cuesta, Debarati Guha-Sapir

**Affiliations:** 1 Centre for Research on the Epidemiology of Disasters, Institute of Health and Society, Université Catholique de Louvain, Brussels, Belgium; 2 Health and Human Development (2HD) Research Group, Douala, Cameroon; London School of Economics and Political Science, UNITED KINGDOM

## Abstract

Hosting refugees may represent a drain on local resources, particularly since external aid is frequently insufficient. Between 2004 and 2011, over 100,000 refugees settled in the eastern border of Cameroon. With little known on how refugee influx affects health services of the hosting community, we investigated the impact of refugees on mother and child health (MCH) services in the host community in Cameroon. We used Cameroon’s 2004 and 2011 Demographic and Health Surveys to evaluate changes in MCH indicators in the refugee hosting community. Our outcome variables were antenatal care (ANC) coverage, caesarean delivery rate, place of delivery and child vaccination coverage; whereas the exposure variable was residence in the refugee hosting community. We used a difference-in-differences analysis to compare indicators of the refugee hosting community to a control group selected through propensity score matching from the rest of the country. A total of 10,656 women were included in our 2004 analysis and 7.6% (n = 826) of them resided in the refugee hosting community. For 2011, 15,426 women were included and 5.8% (n = 902) of them resided in the hosting community. Between 2004 and 2011, both the proportion of women delivering outside health facilities and children not completing DPT3 vaccination in the refugee hosting community decreased by 9.0% (95% Confidence Interval (CI): 3.9–14.1%) and 9.6% (95% CI: 7.9–11.3%) respectively. However, ANC attendance and caesarean delivery did not show any significant change. Our findings demonstrate that none of the evaluated MCH service indicators deteriorated (in fact, two of them improved: delivery in health facilities and completing DPT3 vaccine) with the presence of refugees. This suggests evidence disproving the common belief that refugees always have a negative impact on their hosting community.

## Introduction

In 2015, an estimated 4900 persons were forced to flee their countries every day. Altogether, one in 112 persons in the world today are either refugees, internally displaced, or persons seeking asylum. If this were the population of a country, it would rank as the world’s 21^st^ most populated country. Contrary to common belief, most refugees (nine out of ten) settle in developing countries according to the United Nations High Commission for refugees (UNHCR) [1]. These countries suffer from a range of problems—particularly, they have fragile health systems accompanied by high maternal and child mortality. The additional burden from hosting refugees may further worsen health conditions in the hosting community [2]. Thus, it is common to attribute worsening health conditions to refugees.

In general, risks typically associated with hosting refugees may include disease outbreaks, food and land scarcity, unsafe drinking water, wage competition, overburdened school and health care facilities, and environmental degradation. In extreme cases, refugees are accused of contributing to higher rates of criminality and conflict [3]. On the other hand, the arrival of refugees can enhance the welfare of their host community and stimulate their local economies through higher demand, improvements in infrastructure, and the influx of resources through international humanitarian assistance [4]. Establishing whether the positive effects outweigh negative effects is ultimately a topic of empirical discussion. Many of these mechanisms operate in different ways depending on specific contexts, and the magnitude of their impact is difficult to ascertain as it is inherently difficult to study.

As refugees inhabit an area, assistance programmes to improve their quality of life are rapidly established. Typically, health services capable of addressing mother and child health (MCH) problems are usually established as a priority during assistance to refugees. As a result, the local host population invariably experiences an abrupt improvement in MCH care services in its vicinity. In an optimal assistance program, there should be no disparity in the health care received by the hosts or refugees [4] In keeping with standards, the recommended minimum health services provided in an assistance program usually surpass those experienced by most refugee hosting communities in developing countries [5]. Ultimately, it is expected that the local hosts should experience an improvement in its MCH indicators. Assessing this improvement is critical in guiding recommendations for decision making of the local authorities and assistance programs.

From a policy point of view, one can argue that the impact of refugees on their hosting communities has been neglected for far too long. Fortunately, the UNHCR seems increasingly aware that this overlook is no longer acceptable. The UNHCR now implements new programs to ease the transition phase following camps closure [6]. To this end, there is sound literature evaluating various conditions including health needs affecting refugees [7,8]. But only few studies have addressed the impact of refugees on their host population, and these few mostly focus on economic and security aspects [2,4]. To our knowledge, the impact of the presence of refugees on MCH of the refugee hosting community has not been extensively explored. The absence of an ideal setting where refugees and assistance programmes interact freely with the host population may be partly responsible for this lack of exploration.

Faced with this gap in the literature, the objective of this study is to assess the association between the presence of refugees and changes in MCH services in the refugee hosting community in Cameroon between 2004 (before the influx of refugees) and 2011 (after the influx). Specifically, we evaluate changes in antenatal care (ANC) coverage, caesarean delivery rate, place of delivery and infant vaccination coverage.

## Materials and Methods

### Study Design

We conducted a retrospective secondary data analysis based on two nationally-representative surveys from Cameroon to evaluate the impact of refugee presence on MCH services in a local refugee hosting community. Using data from the 2004 and 2011 Cameroon Demographic and Health Surveys (DHS), we compared MCH indicators of the local refugee hosting community with a control population selected from people living elsewhere in the country. Our control population was selected through propensity score matching. Finally, we performed a difference-in-differences analysis to estimate the effect of the presence of refugees on MCH services in the refugee hosting community. The study was reported in accordance with the STROBE (STrengthening the Reporting of OBservational studies in Epidemiology) guidelines [9].

### Setting

Cameroon is a lower middle income country in sub-Saharan Africa. It has a population of 23.34 million inhabitants spread over 475,650 square kilometers. The country is relatively peaceful and stable [10]. Because of continual conflicts in the neighboring countries, a large number of refugees have been moving to Cameroon. Before 2006, only 4,000 persons of concern to the UNHCR were present in the country. After this period, most Central African Republic refugees fleeing from high level banditry and other criminal acts settled along the eastern borders of the East and Adamaoua regions of Cameroon and spread over an area of more than 50.000 square kilometers. By 2011, the number of refugees had exceeded 100,000 [11].

### Ethical Consideration

Given that this study involved secondary data analysis, the following guidelines were respected: Data were used in strict conformity with the DHS rules and regulations for researchers including sharing data only among registered co-authors, and making no attempt to further identify any individual in the dataset which had been anonymized before access.

### Data Source and Participants

Data were obtained from the DHS programme. The 2004 and 2011 Cameroon surveys included 10,462 and 15,050 ordinary households selected from 467 and 580 clusters throughout the country, respectively. The final survey units (households) were selected through a multistage clustered sampling. The participants selected for our analysis were all women aged 15–49 years, together with their children under one year of age who took part in the 2004 (10,656 women; 425 infants) and 2011 (15,426 women; 501 infants) Cameroon DHS [12].

### Outcome Variables

Our main outcomes of interest were ANC coverage, caesarean delivery rate, place of delivery, and infant vaccination coverage. Access to reproductive health services—including ANC coverage and a skilled birth attendant—are crucial indicators of the continuum of care for mother and child and can be used to assess the change in healthcare delivery [13].

ANC refers to the visits by a pregnant woman to a trained health worker with the goal to detect, treat and prevent pregnancy related problems; the World Health Organisation (WHO) recommends a minimum of four antenatal visits [14]. In the DHS, mothers were asked the number of times they consulted for their pregnancy and then we dichotomized as attendance of less than four ANCs or not. Caesarean delivery was defined as birth through a caesarean section or not. It is estimated that an overall low prevalence of caesarean delivery is an indication of poor quality of maternal and child health services [15]. Place of delivery was defined as delivery in a health facility or elsewhere. Typically, deliveries out of health facilities carry high risks of negative outcomes in these settings. For child care, completing the 3^rd^ dose of DPT vaccine is considered a good indicator for evaluation of vaccination coverage and is widely used in the extended program on immunisation (EPI) coverage [16]. In the DHS, the number of DPT doses the child had received was compared to the child’s age to determine if the child had correctly completed vaccination or not. This variable was therefore defined as non-completion of DPT3 vaccine for children less than one year who had not received their due three doses of DPT.

### Exposure Variable

The exposure variable was the place of residence and was dichotomized as residing in the refugee hosting community or not. Participants were assigned as residing in the refugee hosting community using quantum geographical information system (QGIS) version 2.14. First, clusters in both datasets were redistributed over the national territory based on their GPS coordinates recorded during the cluster sampling phase of the survey. The refugees hosting community was then considered to be the area mapped out by the UNHCR in their 2011 country report [11]. Clusters within the mapped area were considered to be exposed (refugee hosting community).

### Covariates

Our covariates—variables that could predict residence in the refugee zone—initially included the following variables: mother’s educational level (no education, primary, secondary or tertiary education), residence (urban or rural), wealth index (poorest, poorer, middle, richer or richest), mother’s previous birth experience (haven given birth before or not), region of residence (any of the 10 regions in Cameroon), household size, household head (male or female), number children under five years in a household, religion (animist, catholic, muslim, protestants, new religions or others) and ethnicity (any of the 40 ethnic groups reported by participants). Although all the covariates were included in an initial generalized linear model for prediction of propensity scores, we subsequently deleted variables that did not help in the prediction of propensity scores. These variables were likely to generate a difference in our study groups as suggested by the literature [17–19], but they correlated with other variables. For example, a covariate such as wealth index is generated from several other potential covariates (including household’s ownership of selected assets, such as televisions and bicycles; materials used for housing construction; and types of water access and sanitation facilities) which could easily correlate with other variables like household size and household head.

### Statistical Methods

Our exploratory analysis started by evaluating distributions for each numeric variable of interest as well as frequencies and percentages for each of the categorical variables. Specifically, numeric variables were evaluated for normality in their distributions while categorical variables were evaluated for near-zero variation (presence of very few observation in any class) [20]. Graphical displays were used for both univariate analysis and bivariate associations, accompanied by broader tests such as Maximal Information Coefficient [21] and Nonnegative Matrix Factorization [22] algorithms for numeric variables. Missing data were explored using a combination of graphical displays involving univariate, bivariate and multivariate methods.

Propensity scores representing the probability of residing in the refugee hosting community were calculated for each individual, and a one-to-one match [23] based on propensity scores was performed between participants in the refugee hosting community and those living elsewhere using the Matching package in R statistical software [24]. Balance between groups was evaluated through a combination of plots and statistical tests (t- and chi-square-tests). Once matched controls were found, the difference in proportion of each outcome was calculated between the refugee hosting community and their selected controls using the average treatment effect on the treated (ATT). The difference-in-differences of outcomes were then calculated between 2004 and 2011. Both a significance level of p-value less than 0.05 and a 95% confidence interval was used to interpret statistical tests.

## Results

### Characteristics of the study participants

A total of 10,656 women were part of our 2004 analysis, 826 (7.6%) of whom were defined as residents in the refugee hosting community. For 2011, 15426 women were part of the analysis and 902 (5.8%) of them were defined as residents in the refugee hosting community. Considering the 2011 DHS, participants had an average age of 28 years with over 18% of them having no education. Women in the refugee hosting community were on average poorer and had lower levels of education than their counterparts living elsewhere in the country. Also, most women living in the refugee hosting communities dwelled in rural areas and had lesser knowledge on contraception as well as its use. Well over a quarter (27%) of the households were headed by females. The median number of children each woman had given birth to was 2.74 babies, of whom 2.46 were living. The median number of children per household was 1.47 as illustrated in Table 1.

**Table 1:** Background characteristics of study participants stratified by zone of residence (refugee zone), Cameroon DHS, 2004 and 2011.

	2004			2011		
Characteristics	Non-Refugee zone (n = 9830)	Refugee zone (n = 826)	p-value	Non-Refugee zone (n = 14524)	Refugee zone (n = 902)	p-value
Age*	27.48(9.46)	27.65(9.62)	.63	28.01 (9.56)	27.49 (9.45)	0.105
**Education**			<0.001			< 0.001
** No education**	1835 (18.7%)	306 (37%)		2494 (17.2%)	302 (33.5%)	
** Primary**	3945 (40.1%)	362 (43.8%)		5109 (35.2%)	371 (41.1%)	
** Secondary**	3841 (39.1%)	153 (18.5%)		6206 (42.7%)	220 (24.4%)	
** Higher**	209 (2.1%)	5 (0.6%)		715 (4.9%)	9 (1%)	
**Rural dwellers**	4912 (50%)	474 (57.4%)	<0.001	7164 (49.3%)	490 (54.3%)	0.004
Household size*	7.51 (4.68)	7.73 (5.73)	0.285	7.41 (4.49)	7.07 (3.79)	0.008
**Female headed households**	2482 (25.2%)	140 (16.9%)	< 0.001	3912 (26.9%)	189 (21%)	< 0.001
**Wealth index**			< 0.001			< 0.001
** Poorest**	1695 (17.2%)	183 (22.2%)		2075 (14.3%)	217 (24.1%)	
** Poorer**	1638 (16.7%)	188 (22.8%)		2842 (19.6%)	211 (23.4%)	
** Middle**	2113 (21.5%)	209 (25.3%)		2982 (20.5%)	206 (22.8%)	
** Richer**	2120 (21.6%)	134 (16.2%)		3286 (22.6%)	157 (17.4%)	
** Richest**	2264 (23%)	112 (13.6%)		3339 (23%)	111 (12.3%)	
Children under five per household**	1.39 (1.44)	1.66 (1.8)	< 0.001	1.46 (1.45)	1.55 (1.44)	0.07
Children ever born**	2.74 (2.86)	3.09 (3.08)	< 0.001	2.72 (2.77)	3.09 (2.9)	< 0.001
Children living per woman**	2.43 (2.45)	2.69 (2.64)	< 0.001	2.44 (2.38)	2.75 (2.5)	< 0.001
**No child bearing experience**	4454 (45.3%)	334 (40.4%)	0.008	6379 (43.9%)	354 (39.2%)	0.007

* Mean (Standard Deviation).

** Median (Inter Quartile Range).

### MCH Indicators

In 2011, 63.2% of mothers attended the recommended number of ANCs, > = 4. However, mothers in the refugee hosting community were less likely to attend up to four antenatal clinics (43.9% vs 64.6%, p < 0.001). Generally, most deliveries in Cameroon occurred in health facilities (66%), but women in the refugee zone were more likely to be delivered out of health facilities (61.6% vs 32.1%, p < 0.001). Though caesarean delivery rate was less than 5% for the whole country in 2011, it was significantly lower in the refugee zone (4.4% vs 2% p = 0.012). Non-completion of DPT 3 did not show any difference between the two groups in the crude analysis (37.8% vs 40.9%, p = 0.187) as illustrated in Table 2. The difference-in-differences was further calculated prior to propensity score matching and shown in Table 3.

**Table 2:** MCH indicators stratified by zone of residence, Cameroon DHS, 2004 and 2011.

	2004			2011		
Indicators	Non refugee zone	Refugee zone	p-value	Non refugee zone	Refugee zone	p-value
**Number of ANCs attended**			< 0.001			< 0.001
** None**	670 (13.8%)	107 (23.3%)		876 (12.3%)	83 (16.6%)	
** 1 to 3**	1136 (23.4%)	148 (32.2%)		1646 (23.1%)	198 (39.5%)	
** 4 or more**	3043 (62.8%)	204 (44.4%)		4601 (64.6%)	220 (43.9%)	
**Delivery out of Health Facility**	1711 (35.2%)	332 (72.3%)	< 0.001	2286 (32.1%)	309 (61.6%)	<0.001
**Non-completion of DPT3**	1965 (43.5%)	233 (55.2%)	< 0.001	2563 (37.8%)	192 (40.9%)	0.187
**Caesarean Delivery**	117 (2.4%)	2 (0.4%)	0.01	317 (4.4%)	10 (2.0%)	0.012

**Table 3:** Difference-in-Differences of outcomes calculated before propensity score matching.

	2004	2011	
Indicator	Difference in Proportion (95% CI)	Difference in Proportion (95% CI)	Difference-in-Differences % (95% CI)
**Attendance of less than four ANC**	18.3 (13.5, 23)	20.7 (17.2, 25.1)	2.4 (-8.6, 13.4)
**Delivery out of Health Facility**	37.1 (32.7, 41.3)	29.6 (25.1, 33.9)	-7.5 (-20.2, 5.5)
**Non Completion of DPT3**	11.7 (6.7, 16.6)	3.1 (1.5, 7.7)	-8.6 (-16.6, -1.2)
**Caesarean Delivery**	-2.0 (0.9, 2.6)	-2.4 (0.7, 3.4)	0.4 (-4.9, 5.8)

### Testing for Balance after Propensity Score Matching

**Fig 1:**
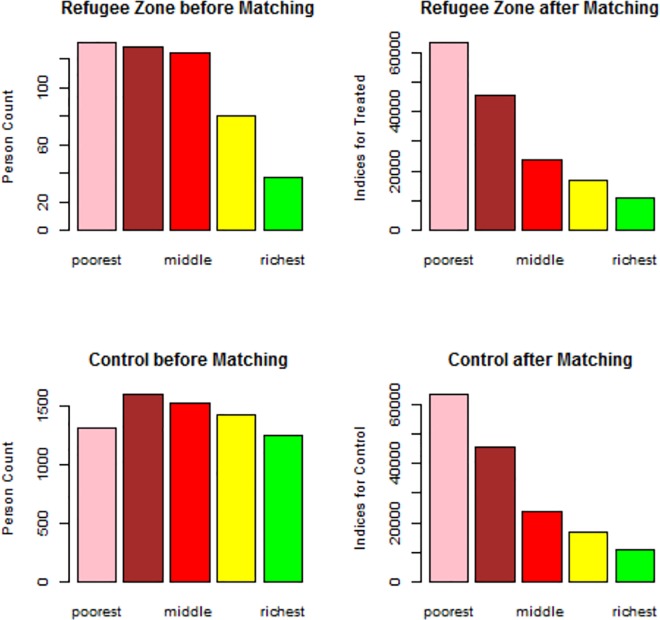
Testing for balance after matching through propensity score distribution by wealth index, Cameroon DHS, 2011.

After the 1:1 matching, each arm had an average of 500 participants for each outcome of interest. Further testing for balance for each covariate included in the model was then performed. Balance was achieved for each of the covariates included as illustrated for wealth index in Fig 1. A table showing the balance for other covariates is included in S1 Table.

### Outcomes after Propensity Score Matching and Difference-in-Differences Analysis

We found that women living in the refugee hosting community in 2011 had a 12.7% (95% CI: 8.5%–17.0%, p < 0.004) higher proportion of non-attendance of the recommended number of ANCs, 17.6% (95%CI: 13.9%–21.3%, p < 0.001) higher proportion of delivery out of health facilities and 1.2% (95%CI: 0.1% to 2.4%, p = 0.078) lower proportion of caesarean delivery when compared to women living elsewhere in the country. On the contrary, after matching, the proportion of children not receiving up to three doses of DPT3 was 2.8% points lower in the refugee hosting community (95% CI: 7.4%–1.8%), though the difference was not statistically significant (p = 0.23) as seen in Table 4.

**Table 4:** Difference in proportions and difference-in-differences of outcomes after propensity score matching, Cameroon DHS, 2004 and 2011.

	2004		2011		
Indicator	Difference in Proportion (95%CI)	P-value	Difference in Proportion (95% CI)	P-value	Difference-in-Differences
**Attendance of less than four ANC**	11.9 (7.3, 16.5)	< 0.001	12.7 (8.5, 17.0)	< 0.001	0.8 (-3.2, 4.9)
**Delivery out of a Health Facility**	26.6 (22.4, 30.9)	<0.001	17.6 (13.9, 21.3)	<0.001	-9 (-14.1, -3.9)
**Non completion of DPT3**	7.2 (2.2, 12.3)	0.005	-2.8 (-7.4, 1.8)	0.23	-9.6 (-11.3, -7.9)
**Caesarean Delivery**	-1.3 (-2, -0.6)	<0.001	-1.2 (-2.4, 0.1)	0.078	0.1 (-4.6, 4.8)

(The difference in proportion is the average treatment effect on the treated calculated as the difference in proportion between the treated and the control. Treated are those in the refugee zone and controls are the matched pairs from the rest of the country)

The proportional differences observed in 2011 when compared to those observed in 2004, the period prior to the large influx of refugees into the country, showed some differences. The proportion of pregnant women not attending up to four ANCs and of having a caesarian delivery remained very similar, while the proportion of deliveries out of health facilities had decreased by 9.0% points (26.6% in 2004 vs 17.6% in 2011). The proportion of non-completion of DPT3 improved by 9.6% points (7.2% in 2004 vs -2.8% 2011) as illustrated in Table 4.

## Discussion

To the best of our knowledge, very few studies have addressed the impact of refugees on the health of their local host population [2,4,25]. This study therefore evaluated how the presence of refugees influenced health services in the hosting community—specifically focusing on MCH. We found that between 2004 and 2011, the attributable change in proportion of women not attending up to four ANCs or undergoing caesarean delivery stayed unchanged while there was a decrease in the proportion of delivering out of health facilities and non-completion of DPT3 for the refugee hosting community.

Generally, the observation of poorer maternal health indicators in the refugee hosting population could be explained by a number of factors. For instance, Cameroon, as other developing countries in sub-Saharan Africa, has poor maternal health indicators as evidenced by the usual high maternal mortality [26]. Though trends in maternal mortality have been decreasing over the last three decades, absolute maternal mortality ratios are still high [27]. These poor maternal health indicators are not evenly distributed over the country since they are influenced by factors that are themselves not evenly distributed. For example, the utilization of health care services, which is an important determinant for maternal health, is itself influenced by several other factors including: educational level, parity, health insurance coverage, ethnicity, household wealth and geographic region [28]. The unequal distribution of these determinants could be expected in our study population as the refugee host population was less wealthy, less educated, had higher fertility rates, and mostly lived in rural areas. Thus, in the first instance, it would be expected that maternal health indicators would be correspondingly poorer.

In addition, the baseline indicators from the 2004 DHS—in other words, before the influx of refugees—demonstrates a pre-existing disparity in MCH indicators. This disparity could also be explained by the fact that our host population was located at the border, and the population at the borders have been found to generally have poorer health conditions [29,30].

However, one could also argue that the presence of refugees in this area could lead to improved health status in the local hosting community. The local host population is invariably exposed to improved healthcare services resulting from assistance programs. In addition, the presence of many refugees in these areas can contribute to social and economic changes. Increased cash and better health services would mean better health indicators.

Among possible determinants of child health, living in a refugee hosting community did not seem to affect children's health negatively when it came to EPI vaccination coverage. The inversion of the risk of low vaccination in the refugee zone in 2011 compared to 2004 may disprove the claim of difficult health access in the refugee zone. The role of assistance programs in putting pressure on the health system to vaccinate children so as to foster herd immunity cannot be denied. Child health programs addressing malnutrition problems in these areas do so without discrimination between resident and refugee children. This may also have helped improve the identification and vaccination of children who would otherwise not have been vaccinated.

The apparent disparity in health indicators between the local refugee host population and women living elsewhere in the country has important implications. It emphasizes the importance of continual evaluation of changes in health parameters (including the movement of refugees), and hence assistance programs in an area. The assessment of the impact of phenomena such as the environment, policy and interventions on the health of a population is however, quite challenging. Different frameworks have been developed to this effect. The Health impact assessment (HIA) framework, for example, is widely used and has the advantage of expressing results in terms of attributable morbidity and mortality [31]. Assessing indicators of morbidity and mortality or their proxies could give a detailed assessment of the health impact of refugees on the host population. The WHO proposes 11 indicators for evaluating MCH [32] and investigating all these indicators would have given a broader and better picture of mother and child health. However, we selected four main process indicators (ANC coverage, caesarean delivery rate, delivery in health facilities and infant vaccination coverage) which we considered sensitive to changes within the time of exposure to refugees.

Despite filling an important gap in the literature, our study does have limitations, most of which are associated with the retrospective cross-sectional design and the use of propensity score matching. First, the entire analysis was based on data in which women report past events. Such data is therefore subject to recall bias. However, because well-trained interviewers were used in this survey, the data collected tend to be of high quality, reliable and free from common errors. Second, we used propensity score matching to select our controls from the general national population, initially motivated by the large number of individuals in the control group. This method only mimics randomization in that it selects controls who had similar chances of residing in the refugee zone based on observed covariates. But other factors that could affect assignment to treatment and outcome that were not observed could not be accounted for in the matching procedure. Also, any hidden bias due to latent variables could remain after matching despite attempts to include the most comprehensive set of covariates. Nonetheless, this method was considered more appropriate since it has optimal matching strategies compared to alternative designs; it improves control of confounding with scarce outcomes; and it has the ability to identify interactions between propensity of treatment and treatment effect on outcome [33]. Third, even though we included caesarian delivery as an outcome—which is acceptable by the WHO as an indicator of quality of maternal and child care delivery in the absence of appropriate data on health service quality—this indicator alone may still not sufficient to comment on the quality of care which is an important aspect of healthcare delivery. Furthermore, the evaluation of the effect of hosting refugees on health services for chronic diseases (including HIV, malaria and tuberculosis as well as non-communicable conditions) that are more complex and costly to manage will yield a more complete picture. Notwithstanding these limitations, the concepts of humanitarian assistance, the interplay between foreign assistance programs and the host government as well as the settling of refugees in less privileged areas are universal. Further multi-country studies might also be able to assess these contextual factors.

## Conclusions

In summary, our findings demonstrate that none of the evaluated MCH service indicators deteriorated with the presence of refugees—in fact, two of them improved: delivery in health facilities and completing DPT3 vaccine. This analysis focused on a specific sample in Cameroon and on basic MCH service indicators. Analyses of more indicators in further settings are required to assess the robustness of these findings. Nonetheless, our study sheds light on the effect of refugees on communities from a healthcare delivery perspective, and suggests evidence disproving the common belief that refugees have a negative impact on their hosting community.

## Supporting Information

S1 Table Balance of covariates after propensity score matching, DHS 2011, Cameroon. (PDF)
