# Supplementary material for: Impact of Refugees on Local Health Systems: A Difference-in-Differences Analysis in Cameroon
Source: PLoS One. 2016 Dec 16;11(12):e0168820. doi: 10.1371/journal.pone.0168820 (PMC5161383; doi:10.1371/journal.pone.0168820)
Supplement: S1 Table — (PDF) [file pone.0168820.s001.pdf]

Variable	Categories	Before Matching			After Matching		
		Treated	Control	Standard Mean Difference	Treated	Control	Standard Mean Difference
**Education**	**No Education**	0.36128	0.22111	29.149	0.36128	0.36128	0
	**Primary**	0.4491	0.39787	10.29	0.4491	0.4491	0
	**Secondary**	0.18164	0.34775	-43.041	0.18164	0.18164	0
**Residence**	**Rural**	0.39721	0.42889	-6.4692	0.39721	0.39721	0
**Wealth Index**	**Poorest**	0.26347	0.18419	17.979	0.26347	0.26347	0
	**Poorer**	0.25549	0.22561	6.8447	0.25549	0.25549	0
	**Richer**	0.15968	0.20048	-11.126	0.15968	0.15968	0
	**Richest**	0.073852	0.17493	-38.609	0.073852	0.073852	0
**Child Bearing Experience**	**No Experience**	0.21557	0.22294	-1.7907	0.21557	0.21557	0
